# Global Critical Care: Moving Forward in Resource-Limited Settings

**DOI:** 10.5334/aogh.2413

**Published:** 2019-01-22

**Authors:** Janet V. Diaz, Elisabeth D. Riviello, Alfred Papali, Neill K. J. Adhikari, Juliana C. Ferreira

**Affiliations:** 1 California Pacific Medical Center, San Francisco, CA, US; 2 Division of Pulmonary, Critical Care, and Sleep Medicine, Beth Israel Deaconess Medical Center and Harvard Medical School, Boston, MA, US; 3 Atrium Health, Charlotte, NC, US; 4 University of Maryland School of Medicine, Baltimore, MD, US; 5 University of North Carolina School of Medicine, Chapel Hill, NC, US; 6 Department of Critical Care Medicine, Sunnybrook Health Sciences Centre and University of Toronto, Toronto, CA; 7 Divisao de Pneumologia, Instituto do Coracao, Hospital das Clinicas da Faculdade de Medicina HCFMUSP, Universidade de Sao Paulo, São Paulo, BR

## Abstract

Caring for critically ill patients is challenging in resource-limited settings, where the burden of disease and mortality from potentially treatable illnesses is higher than in resource-rich areas. Barriers to delivering quality critical care in these settings include lack of epidemiologic data and context-specific evidence for medical decision-making, deficiencies in health systems organization and resources, and institutional obstacles to implementation of life-saving interventions. Potential solutions include the development of common definitions for intensive care unit (ICU), intensivist, and intensive care to create a universal ICU organization framework; development of educational programs for capacity building of health care professionals working in resource-limited settings; global prioritization of epidemiologic and clinical research in resource-limited settings to conduct timely and ethical studies in response to emerging threats; adaptation of international guidelines to promote implementation of evidence-based care; and strengthening of health systems that integrates these interventions. This manuscript reviews the field of global critical care, barriers to safe high-quality care, and potential solutions to existing challenges. We also suggest a roadmap for improving the treatment of critically ill patients in resource-limited settings.

## Introduction

**Figure 1:**
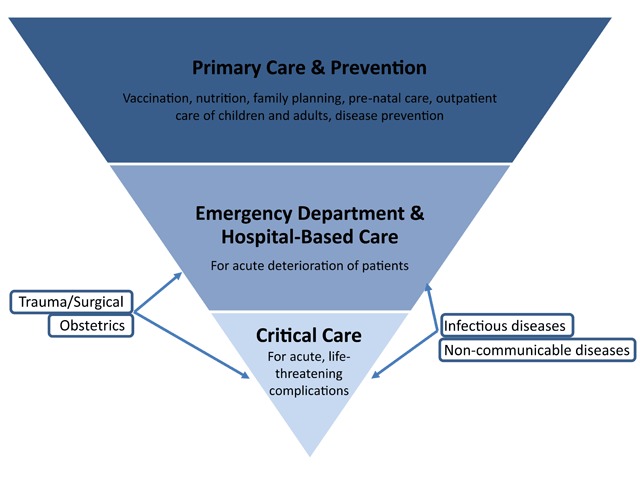
Integrated model of Health System for resource-limited settings. Primary care is the basis of the health system, serving all the population. Other levels of care include hospital care and critical care. All levels are needed for public health response.

Critical care is an important component of health care systems around the world (Figure 1). Caring for critically ill patients in resource-rich settings typically involves treatment in intensive care units (ICUs) staffed with highly specialized health care professionals, systematic monitoring and use of high-cost technology [1]. Unfortunately, these components are not always available in resource-limited settings [2,3], where the burden of disease is greater [4], outcomes are poorer [5,6], and local characteristics require context-specific approaches to the organization of critical care services.

Critical care delivery, education, and research require a global perspective based on epidemiologic considerations. The burden of critical illness in resource-limited settings is not well described, but the best available estimates suggest that it may be greater than in resource-rich settings [7,8] due to deficiencies in access to health care, emergency triage, and lack of early recognition [9,10,11]. Mortality for adults with sepsis [5,12,13] and acute respiratory distress syndrome (ARDS) [14,15,16] in resource-limited settings is higher than in resource-rich settings, and decedents are usually young (mean age 35 years vs. 61 years in United States) [12,17], which contributes to a greater negative downstream social and economic impact. Similarly, there is a higher burden of sepsis and respiratory infection mortality among children [18,19,20,21]. Recent epidemiological changes in global health have created a ‘double burden of disease’ to resource-limited settings [9,22] due to an increase in the prevalence of non-communicable diseases combined with lack of improvement in the long-recognized higher burden of communicable diseases, maternal and child mortality, malnutrition, and human immunodeficiency virus (HIV)-related complications.

Health care professionals from resource-rich and resource-limited settings should take a global perspective on critical care for both ethical and practical reasons. Ethically, health care professionals, trainees, professional societies, non-governmental organizations (NGOs), and international organizations from resource-rich settings have the capacity of deploying resources to improve outcomes for critically ill patients in resource-limited settings [11,23,24]. Assistance can include financial help, knowledge exchange in the form of research and educational partnerships, and capacity building in operations and implementation science. From a practical point of view, supporting resource-limited settings is also important given that inter-related economies, the consequences of pandemics, and conflicts driving mass migration [25,26,27] can all reach far beyond local borders [28,29,30,31].

In recent years, challenges arising from limitations of human, technological, infrastructure, and health system resources in resource-poor settings have been well documented [2,3,9,12,21,22,32,33], but less attention has been given to potential solutions [34,35]. In this article, we review the epidemiology and challenges of critical care in these settings and then focus on potential solutions and opportunities for improvement.

## Challenges to Critical Care in Resource-limited Settings

### Gaps in Epidemiological Data

Comparative epidemiological data on ICU capacity is important for rational allocation of health care resources and to deliver life-saving, cost-efficient intensive care services, especially in settings where resources are scarce [36,37]. However, the burden of critical illness and its global variation are not well established, even by the Global Burden of Disease project [4,9,21,38]. Accurate estimation of ICU outcomes depends on administrative records or representative epidemiologic studies with sufficient follow-up, both of which require a well-organized health care system and a robust research infrastructure that are usually underdeveloped in resource-limited settings. Additional challenges to identifying the burden of critical illness include vague and sometimes varying working definitions of ICUs and critically ill patients [1,39] and inconsistent ICU admission criteria across different settings, usually primarily driven by availability of ICU beds [37,40]. Moreover, the need for critical care is usually substantially underestimated due to barriers to access related to distance, lack of transportation, and cost [22,41]. Population-based estimates of the burden of critical illness in resource-limited settings are lacking [9]. Epidemiologic studies are typically cross-sectional and, except for few studies, have limited longitudinal follow-up to assess survival outcomes [5,15]. There is also limited data on ICU capacity in resource-limited settings [3,33,42], and only a few assessments are population based [12,43,44], precluding accurate national estimates of ICU capacity or the number ICU-treated critically ill patients.

Unfortunately, the standard practice of extrapolating outcomes data from resource-rich to resource-limited settings is misleading. ICU mortality rates in resource-rich settings appear to be decreasing over the last decades [17,45], but this trend is related to case mix, organizational factors, and clinician practices that may be different or inapplicable in resource-limited settings [40]. Thus, improving ICU outcomes globally will require a better understanding of the burden of critical illness and inequities in ICU capacity, with a focus on regional and local data about ICU processes and outcomes in resource-limited settings. An important concept that may guide future epidemiologic estimates is the notion that many patients in resource-limited settings who die of acute reversible illnesses can be assumed to be critically ill and would be offered treatment in an ICU, if it were available.

### Gaps in Evidence for Best Practices

Given that data on treatment outcomes generated by local clinical trials is rarely available, health professionals in resource-limited settings must rely on literature from developed countries. However, interventions that improve outcomes in patients from resource-rich settings may not always be relevant; prominent examples include the higher mortality of septic children and adults treated with aggressive fluid resuscitation in Africa [46,47,48].

Local characteristics require that bundles and protocols developed in resource-rich countries be adapted before implementation in resource-limited settings [49,50]. For example, most sub-Saharan countries lack resources to implement all components of the Surviving Sepsis Campaign Guidelines, but local modification might allow the implementation of specific life-saving interventions [51]. New trials may also inform appropriate modifications to guidelines; for example, routine measurement of central venous pressure is no longer a recommendation in recent Surviving Sepsis guidelines [52], which may improve adherence to sepsis bundles where central venous catheters are not routinely used or available [53].

Mortality prediction scores to risk-adjust in research and quality improvement efforts must also be developed and validated in relevant populations [54,55]. Region-specific equations or adaptations for resource-limited settings can improve performance and facilitate implementation [55,56,57,58,59]. Risk assessment tools, such as the Modified Early Warning Score, which might help triage critically patients and allocate resources [54] have shown conflicting results when applied in resource-limited settings and need additional modifications and validation [60,61].

### Selecting Allocation of Critical Care Resources

Triage decisions for ICU admission are required because demand commonly exceeds supply, even outside pandemics or mass casualty disasters [62,63]. Importantly, these criteria should take into account patient autonomy, which may vary based on cultural and other local factors and should incorporate regional policies. Unfortunately, adequate tools for resource-limited settings are very limited. Even when evidence from resource-rich settings exists, implementation is challenging [65]. Barriers include lack of equipment (e.g., mechanical ventilators to support patients with acute respiratory failure), medications (e.g., antibiotics for multidrug resistant bacteria), diagnostics (e.g., equipment for repeated measurements of lactate and blood gases to guide treatment of sepsis or ARDS), trained personnel, and quality improvement systems. Therefore, clinical trials of novel therapeutic approaches and implementation strategies are required to ensure the ‘right solution for the right setting’.

## Potential Solutions and Opportunities for Innovation

While challenges are formidable, many opportunities exist to improve critical care in resource-poor settings in the categories of ICU organization, clinical guidelines, education, research capacity, and health care system strengthening.

### ICU Organization

The World Federation of Societies of Intensive and Critical Care Medicine (WFSICCM) developed a common framework describing critically ill patients and critical care service, targeting policy-makers, clinicians, and patients, which has led to several consensus-based documents [1,39,62]. The task force proposed a three-tiered system of categorizing ICUs into: 1) level 1 units, capable of providing oxygen, noninvasive monitoring, and more intensive nursing care than a regular ward; 2) level 2 units, which have short-term ability of providing invasive monitoring and basic life support; and 3) level 3 units, capable of providing the full spectrum of monitoring and life support technologies, serving as a regional resource for the care of critically ill patients, and also playing an active role in research and education [1]. With these standardized definitions, different countries can create a more accurate inventory of their ‘ICU capacity’ allowing for meaningful comparisons across regions. In addition, the Global Intensive Care Working Group of the European Society of Critical Care Medicine has developed recommendations for infrastructure and ICU organization in resource-limited settings [64].

The WFSICCM guidelines also described the training needs, roles, and responsibilities of critical care specialists [39] and the need for uniform training pathways to ensure minimal standards and competencies. The report recommends adoption of existing competency-based training frameworks, such as the Competency-Based Training in Intensive Care Medicine program in Europe (CoBaTrICE) and the CanMEDS competencies of the Royal College of Physicians and Surgeons of Canada. These documents delineate competencies related to medical knowledge –such as diagnosis, monitoring, and treatment of patients with critical illnesses – and leadership, professionalism, end-of-life care, and communication, among others.

Finally, the WFSICCM task force emphasizes the importance of intensivist-led triage based on input from other clinicians and following institutional or regional policies, regardless of patients’ socioeconomic status, insurance coverage or ability to pay for care [62,63].

### Education and Capacity Building

Educational interventions to build critical care capacity in resource-limited settings are important because of the underrepresentation of relevant topics in medical school curricula, the lack of post-graduate, critical care training pathways, and limited access to continuing medical education [66,67]. Interventions can range from short focused courses to longer-term programs based on academic partnerships between high- and low-income countries (including academic institutions, professional societies, and NGOs) that aim at training critical care specialists [66,67]. Short courses (Table 1) cannot replace long-term clinical training but have the advantage of providing standardized education on specific topics to many clinicians, and can be implemented in response to acute needs. However, the impact of short courses on longer-term knowledge, bedside processes of care, and patient outcomes has not been well studied.

**Table 1:** Examples of Short Critical Care Courses Available in Resource-limited Settings.


The Basic Assessment and Support in Intensive Care (BASIC) course, Chinese University of Hong Kong [70]	This is a short, intensive course that is widely available and free, with standardized training material that covers a broad range of intensive care topics [66]. Its target audience includes critical care physician trainees, critical care nurses, non-intensivist physicians, and allied health workers. Dissemination has relied on a train-the-trainers approach, with cascade trainings within countries by local facilitators.
World Health Organization short course on the critical care management of Severe Acute Respiratory Infection (SARI) [71]	This course is designed to respond to outbreaks of severe respiratory infections and has trained over 1000 intensive care unit doctors from 13 countries, focusing on clinical management of patients with severe pneumonia, sepsis, and acute respiratory distress syndrome. It is a collaborative effort between local health authorities, the WHO regional offices, and the WHO expert clinical network.
Network for Intensive Care Skills Training (NICST), Sri Lanka	NICST is a non-profit, international organization that provides training for critical care nurses [72,73] in Sri Lanka with a train-the-trainers approach. An assessment found improvement of immediate knowledge and high levels of participant satisfaction [74].
American Thoracic Society (ATS) Methods in Epidemiologic, Clinical and Operations Research (MECOR) course	This series of courses is sponsored by the ATS and local respiratory societies and has trained over 1000 health professionals in 24 countries in research methodology [75].


In contrast, longitudinal academic partnerships (Table 2) can provide knowledge exchange platforms and build local ICU faculty expertise, but these depend on continuous on-site mentorship and training by visiting international faculty. Important factors for sustainability and avoidance of redundancy include integration with local medical education systems (pre- and post-graduate), alignment with priorities of local health authorities, development of academic partnerships in the country or region of interest [67], and adaptation to local cultural values [68,69]. In addition, it is essential to have a secure source of funding to ensure programmatic sustainability, retention of newly, highly trained faculty and continued expansion. Finally, these partnerships should follow WFSICCM uniform training recommendations for ICU specialists to ensure minimum standards and competencies necessary to practice effectively in their environment.

**Table 2:** Examples of Academic Partnerships.


**The East African Training Initiative:** A two-year fellowship program in pulmonary and critical care medicine hosted by the Tikur Anbessa (Black Lion) Teaching Hospital of Addis Ababa University (AAU) School of Medicine in Ethiopia. This is a collaborative effort between the health ministry, the AAU, international faculty, the World Lung Foundation and the Swiss Lung Foundation [69] and had graduated nine specialists as of January 2019.
**The Rwanda Human Resources for Health (HRH) Program:** A partnership between 25 American institutions and the Rwandan Ministry of Health. It aims to increase capacity to 500 specialist-trained physicians within the seven years of its funding and includes critical care [76].


### Research

Opportunities for research in epidemiology, diagnosis, therapeutics, and implementation of critical care resources in resource-limited settings are vast. We outline areas of investigation with promising studies completed.

#### Burden of Disease

The Global Burden of Disease project, which has the goal of identifying risk factors and estimating the health impact of different diseases [77], does not directly address critical illnesses. For example, lower respiratory infections remain the leading cause of death in children less than five years of age in low-income countries [77]; however, number of deaths due to respiratory failure and sepsis remains unknown. As a result, insights into critical care epidemiology have relied on convenience samples of ICUs with more recent studies including more participants from low- and middle-income countries [5]. However, because most septic patients are not admitted to an ICU in resource-limited settings, methods of incidence estimation that rely on ICU-treated cases underestimate morbidity and mortality. Another approach to understand sepsis-related mortality would be to examine causes of death based on interviews of relatives using verbal autopsy methods, as done for surgical conditions and renal failure [78,79].

#### Early Recognition and Treatment of Critical Illnesses

In resource-limited settings, developing tools and interventions for early detection and treatment of critical illness could prevent multi-organ failure and death and ease burden on limited ICU resources. In one ICU in Tanzania, an observational study showed that detection of vital sign derangements infrequently led to treatment modifications [80] and led the authors to conclude that nursing-based vital signs–directed clinical response protocols may not improve outcomes in resource-limited setting. Early warning systems have been shown to be predictive in some [54] but not all settings [60,61], underscoring the need for further development and validation in resource-limited settings.

Globally, ARDS remains under-recognized and under-treated [81], particularly in settings where a consensus definition cannot be readily applied due to the limited availability of arterial blood gas analysis, chest radiography, and positive pressure ventilation. To overcome this challenge, investigators developed modified ARDS criteria using oxygen saturation/inspired fraction of oxygen ratio and lung ultrasound and applied this classification to estimate incidence and mortality of ARDS in one hospital in Rwanda [14]. Such modified criteria, after additional validation, could be used to enhance our understanding of epidemiology and design clinical trials.

Septic shock also remains a major cause of mortality in adults and children globally [82], and it is clear that early recognition and treatment save lives [83]. Increasing attention to this condition has grown after the establishment of the World Sepsis Day [84] and the related 2017 World Health Assembly resolution [85]. However, trials of fluid resuscitation in sepsis in sub-Saharan Africa showed increased mortality in children [46] and adults [47,48] illustrating the challenges of applying well-accepted resuscitation algorithms from resource-rich settings (that assume mechanical ventilation availability and intensive care capacity) in resource-limited settings. Defining safe fluid resuscitation approaches for resource-limited settings is a major research priority and reinforces the need for clinical trials that include rules for stopping therapy and that minimize risk of adverse effects by integrating local ICU practices [86].

#### Quality Improvement and Behavior Change

Quality improvement (QI) is an essential tool for making health care safer and more effective and another promising research area for resource-limited settings. Due to local site practices, there can be substantial differences in the effectiveness of QI interventions. Prominent examples are from Brazil, and include a before-after study of a multi-faceted QI intervention including screening strategies, multi-disciplinary educational sessions, case management, and continuous performance assessment, resulting in improved adherence to the Surviving Sepsis Campaign 6h-bundle and lower mortality [87]. A cluster randomized trial in 118 Brazilian ICUs compared routine care to a another multi-faceted QI strategy that included a daily checklist, goal setting during multidisciplinary rounds, and clinician prompting for 11 care processes and found no effect on mortality and variable impact on processes of care [88]. Another ongoing project is testing a checklist for early recognition and treatment of acute illness (CERTAIN), a decision support tool for initial and follow-up management of critical illness syndromes currently under evaluation in resource-rich and resource-poor settings [89]. Conducting local QI projects in resource-limited settings, using small samples to test changes in outcomes and measuring impact on processes of care, is essential. Sharing successes and failures can inspire colleagues to test innovative approaches to behavior change leading to improved implementation of evidence-based interventions.

#### Ethics

The requirements for conducting critical care research in resource-limited settings need improvement to enable timely completion while ensuring protection of the rights of research participants and academic recognition to investigators in low- and middle-income countries. Unfortunately, currently prevailing models prioritize ‘blockbuster products’ that do not necessarily recognize a health rights framework or public health approach, and grossly fail to address population needs, especially for resource-limited settings, and are poorly suited for timely response to outbreak conditions [31]. Folayan et al. describe four major processes required to conduct research in resource-limited settings during outbreak conditions, including local access to products developed as a result of the research, capacity transfer to local researchers, development of competent local ethics committees, and empowerment of community members to actively engage in research design and implementation [90]. Public health organizations should work with the academic community to guide research priorities by taking into account the public health impact. In response to previous global health emergencies, the World Health Organization (WHO) and the International Severe Acute Respiratory and Emerging Infection Consortium (ISARIC) have collaborated to develop modular standard case report forms that can be used in multiple settings to promote faster data collection during infectious disease outbreaks [91]. A severe acute respiratory infection (SARI) observational study is being conducted globally in collaboration with ISARIC, the International Forum of Acute Care Trialists and the Platform for European Preparedness Against (Re-)emerging Epidemics to test the global research response capacity, estimate global SARI incidence, and understand barriers to the research processes [92].

### Clinical Guidelines

Until sufficient research from resource-limited settings drives locally generated clinical guidelines, adaptation of existing guidelines [52,93,94] is essential to bring safe, feasible, and effective practices to the bedside. For example, the European Society of Intensive Care Medicine Global Health Working group, including experts from both resource-rich and resource-limited settings, developed adapted recommendations for the management of sepsis in resource-limited settings. Topics covered include ventilatory support [95], sepsis recognition [96], and sepsis management in adults [20] and children [19]. These papers highlight the lack of primary evidence from resource-limited settings and advocate that future guidelines should be based on locally produced evidence and use GRADE-adherent processes. Similarly, the WHO has published clinical management guidelines for severely ill children [97,98,99], adolescents, and adults [100] in austere environments and more recently for dengue [101], malaria [102], and viral hemorrhagic fever [103]. Although these guidelines do not include mechanical ventilation or other ICU technologies, they do address triage and emergency treatments. Implementation of pediatric triage has been associated with improved outcomes in Sierra Leone [104], and a single center, pilot study in Haiti evaluating the Integrated Manual for Adult and Adolescent Illness (IMAI) protocol for severe sepsis demonstrated increased sepsis recognition, greater volume of fluid resuscitation, and increased frequency of vital signs monitoring [105].

### Strengthening Health Systems

To improve critical care service delivery in resource-limited settings, decision-makers must accept that high-quality, equitable intensive care services are necessary to achieve the Sustainable Development Goals and health security [34,106]. These services should include basic hospital resources, a reliable supply chain for essential medications and equipment [107], and a plan for human resource development [1]. In resource-limited settings, decision-makers include local health authorities (often supported by the WHO), international donors, and NGOs. Strengthening the health system in these settings should include service delivery, education, and research on quality improvement, comparative effectiveness, and cost-effectiveness practices. This requires not only ‘building bridges’ among partners, but also creating a common vision. Finally, critical care must be organized and integrated into the larger health care system, including pre-hospital emergency medical services, emergency department care, ward-based care, and surgical and obstetrical care, so that critically ill adults and children are recognized at any location and treated with prompt, appropriate life-sustaining interventions [108].

## Conclusions

Caring for critically ill patients in resource-limited settings is challenging due to the high burden of disease and high mortality rates from potentially treatable critical illnesses. Despite the lack of epidemiologic data, deficiencies in health systems organization and resources, and institutional obstacles to implementation of effective interventions, many potential solutions are emerging. We suggest the following roadmap for the improvement of care for critically ill patients in resource-poor settings:

1. International organizations must recognize that the delivery of safe, equitable, and high-quality critical care in resource-limited settings is a priority for international health security.
2. Education and research activities must be integrated into national healthcare priorities.
3. Critical care education should be expanded in a uniform, sustainable fashion, including short courses to improve general knowledge as well as comprehensive, competency-based specialty training.
4. Research in resource-limited settings must take into account the public health impact and be prioritized to include epidemiologic investigations of burden of disease and access to critical care, evaluation of diagnostic and therapeutic interventions, quality improvement, and cost-effectiveness.
